# Clinical and economical improvements after introducing rapid identification of bacteria and early antibiotic susceptibility testing in sepsis and bloodstream infections. Results of the PHENOMENON study

**DOI:** 10.3205/id000069

**Published:** 2020-12-15

**Authors:** Michael Wilke, Wolfgang Heinlein, Luis Stiefenhofer, Klaus-Friedrich Bodmann

**Affiliations:** 1 inspiring-health GmbH, Munich, Germany; 2 Kliniken Nordoberpfalz AG, Klinikum Weiden, Germany

**Keywords:** rapid bacteria identification, antimicrobial susceptibility testing, AST, sepsis, bloodstream infections, antimicrobial stewardship, health economics, costs

## Abstract

**Background:** Sepsis and bloodstream infections pose severe challenges in intensive care. Early reliable diagnosis is the key to successful therapy. The objective of the study presented here was to investigate the clinical and economical effects of the new Pheno^TM^ BC test, which allows bacteria identification (ID) and antimicrobial susceptibility testing (AST) in approximately 7 hours after a blood culture becomes positive (BC+).

**Methods:** Historically controlled interventional study. Population: patients with BC+ and ICU admission. Inadequate initial antimicrobial therapy (IAT) is need of therapy change based on result. Prospectively the new test was used in addition. Primary endpoint: time-to-result in hours. Contribution margin (CM) i.e. revenue – costs was computed. All patients formed the intention-to-treat population (ITT). Patients with complete cost data formed the modified ITT group (mITT). CM results were calculated for mITT and PP. Further analyses: length-of-stay (LOS) and mortality.

**Results:** 223 historical and 200 prospective patients were included. Time to result (ITT) was shortened by 51.1 hours (83 vs. 31.9; p<0.001). Overall savings (mITT) were 257,100 € (–301,264 € vs. –44,164 €). 143 of 181 (79%) patients had a test performed, 126 of 143 (88%) having a clinically useable result. 40 (32%) had IAT vs. 65 (29%) in the historic cohort. Median time to AST in PP was shortened by 61.7 hours (89.5 vs. 27.8; p<0.001). LOS was shortened 7 days (28 vs. 19; p=0.226) and mortality was 8% (40.5% vs. 32.5%; p=0.440) lower. Median CM +3,074.80 € per case (–2,350.50 € vs. +724.70 €; p=0.040).

**Conclusion:** The new Pheno^TM^ ID+AST test leads to faster and clinically meaningful results and saves money by shortening LOS on the ICU.

## Background

The treatment of sepsis and bloodstream infections in the intensive care unit (ICU) is time critical. Inadequate initial antimicrobial treatment (IAT) leads to longer length of stay (LOS) and worse outcomes [1], [2]. A number of studies have already shown that the rapid identification (ID) and antibiotic susceptibility testing (AST) are key technologies to achieving adequate antimicrobial therapy (AAT), with a shorter LOS and less deaths in the hospital [3], [4]. Approximately one patient over four receives IAT [4], [5]. An analysis of the recent literature by the authors resulted in an average of 25.4% IAT in sepsis and bloodstream infections (9%–63%) [2], [5], [6], [7], [8], [9]. The average LOS in hospital was 4.6 days longer, when patients received IAT. Antimicrobial stewardship programs (ASP) have shown improvements in prescribing practice, with reducing IAT rates [10], [11]. Reliable ID and AST are key factors in all ASP [12].

Since a few years, a new technology that delivers ID and AST within 7 hours after positivity of a blood culture (BC+) is available. The Pheno^TM^ system provided by Accelerate Diagnostics, Tucson, Arizona, USA, provides reliable results in monobacterial infections and can be used as add-on to existing testing strategies [13], [14], [15], [16]. The system uses fluorescence in situ hybridisation (FISH) probes for ID, and analyses the growth of bacterial colony forming units (CFU) with a high-definition microscope. The analysis of the digital images is translated into growth curves from which – knowing the exact antibiotic concentration in each growth chamber – the minimal inhibitory concentration (MIC) for each antibiotic agent can be calculated. Detailed explanations on the technology have been published [17], [18], [19]. 

The objective of this study was to measure time gains in AST results and to evaluate the economic impact of the new testing strategy. The early change in antimicrobial therapy (AT) is presumed to be the key driver to achieve savings [20], [21].

## Methods

The PHENO Medical EcoNOmic EvaluatioN – PHENOMENON study was conducted in a tertiary care hospital in Germany. 

The study is a historically controlled interventional study comparing the time to result and the economic impact of the new fast ID/AST test with the results achieved by standard-of-care (SoC) in patients with sepsis or bloodstream infection that had BC+ and were admitted to the ICU. The basic assumption was that the new test can provide results faster, enable earlier therapy adjustment and therefore is economically favourable. In the hospital where the study was conducted, every BC drawn was processed in the 24/7 lab until positivity. Positive BC (BC+) are sent to the microbiology lab, which is approximately 80 km away and does not operate 24/7. In this lab, an ID by MALDI-TOF is processed and AST is obtained with the VITEK^®^2. Once the complete AST is ready, it is transferred via electronic data connection into the hospital information system (HIS). In the prospective cohort, positive BC were processed with the Pheno^TM^ system in the 24/7 onsite lab and then sent on to the microbiology lab for comparison purposes and to maintain the current SoC. The Pheno^TM^ test uses 5 ml of blood from each bottle and hands on-time of the system is 2 minutes [14]. In both groups, the antimicrobial therapy (AT) was reviewed by two experienced stewardship experts and separately by an independent reviewer. 

The new test delivers ID in 60–90 minutes, AST after 7 hours. Results with either *too few cells* (e.g. in patients already receiving AT) or *off-panel* (pathogen detected yet not identified) were rated *not clinically useable*.

The historic review was performed in Q1 2018 on cases from 2017. Case selection through the BC+ (data from microbiology lab) and admission to ICU. Prospective arm of the study was from July 1^st^, 2018 to June 30^th^, 2019. Data were collected after four weeks, then quarterly, and patient records were reviewed. In total, five reviews were undertaken. The independent reviewer reviewed all cases after finalization of the study.

The review of the AT in BC+ ICU patients focused on the following assessment: Is the result of the blood culture clinically relevant, i.e. is the pathogen detected relevant for clinical management? If not, it was rated as “contamination/no clinical relevance”. If coagulase negative staphylococci were found and only one bottle was BC+ (standard sample is 4 bottles), the result was rated as contamination/no clinical relevance [22]. In contrary, if the pathogen was detected in two bottles from different locations or the medical record gave clear hints that the clinicians rated the finding as relevant, AT was evaluated.

AT was classified as *adequate* if it is in line with the ID and AST results and no change is necessary. *IAT* was assigned if pathogen was resistant against the current antibiotic regime, therapy change (mono or combination therapy) is necessary or in case of fungal infection, an antimycotic therapy is necessary or earlier start of antibiotics possible in situation where antibiotic pause was performed. *De-escalation* (narrowing of the AT spectrum) was another result of the assessment. If the medical record was incomplete, the assessment was *evaluation* *not* *possible*.

The primary clinical endpoint of the study was the time to result compared between historical and prospective cohort and calculated as difference in median hours between the both respective groups. Time to result is an accepted measure for the efficacy of diagnostics and has impact on patient outcomes [23], [24], [25]. For the historical cohort, the time stamps from the database of the microbiology lab were used. Time was measured from BC drawn to transfer of final AST result. In the prospective cohort, all patients that had at least one BC+ and were admitted to the ICU built the intention-to-treat (ITT) population. Time was measured from BC drawn to the time of transfer of the Pheno^TM^ result to the ICU (by fax). Earlier studies in England already showed a positive effect on the time to result and a number of changes in therapy [26]. As the clinical and microbiology lab setting in the hospital was different and German hospitals are under constant time and financial pressure due to the rigid DRG payment scheme, we decided to use the same primary endpoint. To our knowledge, PHENOMENON was the first study that aimed a proof-of-concept in a clinical routine setting in Germany.

For the economic analysis, the patients from the ITT population built the basis. Cases with missing data (e.g. no cost data) or that were not discharged at end-of-study were excluded. All other cases were assigned to the modified ITT (mITT) population which was used to calculate the overall financial result for the hospital. From the mITT population the test adherence – for assessing how many of these patients received a test – was calculated. From the number (no.) of patients in mITT that received a test and the no. of clinically useable tests, the clinical usability rate was calculated.

All historical and prospective patients among the patients with a clinically useable test, who were found to have IAT, were considered as the most likely to economically profit from the new test. They built the per-protocol population (PP).

The primary economical endpoint was contribution margin (CM) in PP. Costs were calculated out of the German DRG cost matrix using the hospital individual costs that have been retrieved from the controlling department. Revenue is the total DRG payment (including eventual “Zusatzentgelte” (ZE)). CM = revenue – costs of each individual patient. CM as measure of economic success is established in hospital controlling and long in use [27], [28], [29]; secondary endpoints were mortality and total LOS in hospital as they are related to IAT [5], [6], [30].

Table 1 (Tab. 1) gives an overview on the study design following the PICO principle.

The following possible confounders were identified:

- Age, gender
- Reason for admission
  - For German DRGs: primary diagnoses by ICD-10 chapter
- Morbidity
  - Charlson Comorbidity Index (CCI)
  - DRG-CCL value of all secondary diagnoses (SUM_CCL)
- DRG partition
  - Surgical
  - Other
  - Medical
- Treatment intensity
  - ICU days
  - Proportion of mechanically ventilated patients
  - Hours of mechanical ventilation

Before comparison of the results in the populations, an analysis of the possible confounders was performed to check whether one or more of them are statistically significantly different in the two groups. In case of such a difference, a propensity score matching would have been conducted to control the influence of the confounders.

Statistical analyses were performed with SPSS version 19. As all variables were not normally distributed, median and interquartile ranges (IQR) were computed and used for the statistical tests. For continuous variables the Mann-Whitney-U test and for categorial variables either Chi-square or Fisher’s exact test were performed.

## Results

In the historic cohort, we identified 784 patients with BC+ of which 223 were on ICU. In the prospective cohort, 812 patients had BC+ and 200 have been admitted to ICU. Of the 200 patients, 181 had complete data. 19 patients were excluded because they were either not yet discharged or had missing cost data. Table 2 (Tab. 2) shows the patient populations.

Time to result improved by 51.1 hours (83 vs. 31.9; p<0.001) hours median time in the ITT population. The overall financial result for the hospital (mITT) showed an improvement in total CM of 257,100 € (1,107 € per patient). The new test was performed in 143 of 181 mITT patients. The overall adherence to the testing protocol was 79%. The clinical usability was 88% (n=126/143). In the historical cohort, 65 patients (29%) had IAT, and in the prospective cohort 40 patients (32%) needed an adjustment of their antimicrobial therapy due to inadequate treatment. Table 3 (Tab. 3) shows the overall results of the AT assessment in all cases.

The group of patients with IAT (per-protocol population) were assessed by direct comparison. First the baseline characteristics were analysed to make sure that no confounders on the results – especially the economic evaluation – were missed.

Table 4 (Tab. 4) shows the baseline characteristics of the two groups (PP).

As the baseline characteristics showed no significant differences between the groups concerning the pre-defined confounders, the endpoints could be directly computed. The propensity score matching of historical and prospective cases was not necessary.

The primary endpoint was time to AST which was reduced by 61.7 hours in median (89.5 vs. 27.8; p<0.001). The total hospital stay (LOS) was reduced by 7 days (28 vs. 19; p=0.226) and mortality by 8% (40.5% vs. 32.5%; p=0.440). The contribution margin significantly improved by a median value of 3,074.80 € per case (–2,350.50 € vs. +724.70 €; p=0.040).

Table 5 (Tab. 5) shows the results in the per-protocol population.

The total economic result in the PP population was calculated by using the CM in the historical cohort, the CM in the prospective cohort and the test costs incurred for the prospective cohort. CM improved by 289,225 € in total (4,822 € per patient; arithmetic average). 

Details on financial results see Table 6 (Tab. 6).

## Discussion

The findings in this study are in line with those of other authors. Rapid ID and AST significantly shorten the time to result [20], [31], [32]. The gain of more than 2 days is partly explained by the fact that the hospital where the study was performed has no onsite microbiology laboratory. It is located approximately 1 hour away from the hospital. Moreover, this lab does not perform microbiology 24/7. It is important to mention again that the time is the total time from BC drawn to AST result transferred to the ICU. However: Kidd et al. also reported a gain of 41 hours to AST (this study 50 hours) where the lab is onsite [26]. A time gain of 2 days seems to be realistic for the new test compared to SoC considering the local setting.

The savings show a significant increase in contribution margin. This is due to the reduction in overall LOS in hospital of 7 days. The reduction is high but in line with the findings in other studies which were investigating the effects of rapid diagnostics in bloodstream infections and sepsis. Perez et al. found a reduction of 8 days in their study on bacteraemia due to gram-negative pathogens [20]. They also did a saving analysis which resulted in savings of 26,298 US$ per patient. The different costs in the US healthcare system do not allow to relate these numbers to our median savings of 3,074 € per case. Bilir et al. found a reduction of 8.8 days in candidemia patients [33]. Galar et al. found 2.3 days using the same testing modality (VITEK^®^2) and comparing patients where results were available either on the same day or not [21]. Time gains and improvements in contribution margins (CM) are not normally distributed, thus the median time and median CM were used instead of the arithmetic mean. Test costs of 195 € per patient were used for the new test. Total costs of the test from the hospital perspective include also the procurement and maintenance of the machines which are not covered with the DRG payment anyway. We did not add these costs in order to stay in the methodology of the G-DRG. Hospitals that want to perform a total cost of ownership calculation can take these additional costs into account but should also add “capital” costs to all other areas like daily costs, radiology and others. Depending on the purchase model (buying the machine, leasing or reagence rental) the total test costs of the test are 238 € up to 297 €. Applying total costs without taking other capital costs into account, the new test still leads to a positive change in CM.

The PP population was drawn from the number of patients with IAT. The rate of 29% and 32%, respectively, are in line with findings in other studies on sepsis and bloodstream infections [4], [5], [7], [8]. A recent meta-analysis undertaken by the authors showed an average IAT rate of 25.4%. IAT clearly has known and proven economic impact [2], [5], [6], [9]. De-escalation is a strategy under discussion [34]. A recent review concludes that it doesn’t have any effects on costs or LOS [35]. Many opportunities to deescalate are missed [36]. Due to these findings, patients with a chance to de-escalate AT were excluded from PP. Moreover, patients receiving already AAT were also excluded as they had no expectation of savings due to better therapy. We also excluded the patients where the test only showed contamination which was neither influencing the therapy scheme. This is conclusive with findings in the literature [22], [37].

In studies that are designed as before and after cohort studies like ours, effects can be caused by other factors than the study intervention. Although no significant changes in ICU management of the patient groups in the focus of this study were known and the groups do not differ in their relevant baseline characteristics, we performed a regression analysis to assess the influence of the new test and the faster switch from IAT to AAT. The new test turned out to be an independent positive influencing factor on CM. In a linear stepwise regression model, we tested the influence of ICU treatment, mechanical ventilation and the new test on CM. Only the new test showed a significant positive influence (regression coefficient=5.304; std. error= 2.124; T= 2.498; p=0.014) the other variables have been excluded by the program. This clearly shows that the intervention has significant influence on the financial outcomes of the patients. The logistic regression showed an increased probability of survival which was yet not significant (OR=1.385; 95% CI: 0.606–3.166; p=0.441). A bigger sample would be necessary to analyse whether or not there are significant differences in survival. We think it is a positive signal which needs further investigation.

The utility of rapid ID and AST is bound to the timely change in AT following the results. Perez et al. showed that the combination of introducing rapid ID (through MALDI-TOF) plus direct communication of the results is key for time savings [20]. Of approximately 70 hours in shortening the time to change of therapy, 46 hours were due to the direct reporting of the results via telephone. We analysed that the results, which were automatically transferred to the ICU in the moment the Pheno™ result was ready, were turned into change of prescription (if necessary) in less than two hours in average. Studies that examined the single effect of stewardship programs also showed that LOS can be shortened and the quote of adequate antimicrobial therapy is increasing [10]. Pathogens and resistance genes can be quickly identified with low cost methods like MALDI-TOF and in some studies this already leads to savings [20], [31]. In our study we focussed on the effects that can be generated by technologies that provide a complete AST result in short time. New fast AST directly from BC+ also deliver fast results on very moderate costs but still need expert knowledge from the microbiology lab which is usually not available 24/7 [38]. Probably the combination of methods is the best way to achieve a balance in additional costs and achievable savings.

Some publications propose that effects of rapid ID and especially rapid AST are particularly improving antimicrobial therapy of infections caused by Gram-negative bacteria (GN) [14], [31]. The role of Gram-positive bacteria (GP) are considered as less important. Fungal infections (FI) play a certain role. In our study, we found that GN accounting for 56% of all changes recommended by the system, GP for 35% – here MRSA with IAT only covering MSSA was the most frequent – and FI accounted for 9% of the proposed changes. This study confirms that GN are most important. However, MRSA is still an issue and should not be underestimated.

## Conclusions

The introduction of the new rapid ID/AST test allows optimization of AT 2 days earlier, to reduce LOS in the hospital by 7 days and to significantly improve the financial result in patients with IAT. The test is a valuable tool for patients with sepsis and bloodstream infections in ICU. The total savings clearly overcompensate the additional test costs. Early signals for an improvement in survival should be verified in studies appropriately powered for this endpoint.

## Abbreviations

- AAT = adequate antimicrobial therapy
- ASP = antimicrobial stewardship program
- AST= antibiotic susceptibility test result
- AT = antimicrobial therapy
- BC = blood culture
- BC+ = positive blood culture result
- CFU = colony forming units
- CM = contribution margin (in €) = revenue – costs of an individual patient
- FI = fungal infections
- FISH = fluorescence in situ hybridisation
- GN = Gram-negative bacteria
- GP = Gram-positive bacteria
- IAT = inadequate antimicrobial therapy
- ICU = intensive care unit
- ID = identification (of pathogens)
- ITT = intention to treat population
- LOS = length-of-stay (in days) in hospital
- MIC = minimal inhibitory concentration
- mITT = modified intention to treat population
- PP = per-protocol population
- SoC = standard-of-care testing
- USA = United States of America

## Notes

### Ethics approval and patients’ consent

An ethics approval was not necessary, a CE-marked licensed diagnostic test was introduced in clinical routine. No extra intervention like additional blood samples were undertaken. All requirements of the German “Datenschutzgrundverordnung” (DSGVO) were met. Data analysis and patient record review was conducted under a contract for “Auftragsdatenverarbeitung” (ADV) between researchers and hospital. The analysis of laboratory results and antimicrobial treatment was covered with the patients' general consent given in the treatment contract with the hospital that entitled the hospital to analyse data for scientific and quality assurance purposes. Consent for publication was not necessary since no individual reports are reported.

### Availability of data and materials

The datasets used and/or analysed during the current study are available from the corresponding author on reasonable request.

### Competing interests

MW as CEO and CMO of inspiring-health received research grants from Accelerate Inc., Pfizer, Beckton Dickinson and Norgine. He received honoraria for lectures and travel cost coverage from Accelerate Inc., Beckton Dickinson, Correvio, the German Society of Hospital Hygiene (DGKH), the German Union of Internal Medicine (BDI), Pfizer, and ROCHE.

WH and LS are employees of inspiring-health.

KFB received honoraria for lectures and travel cost coverage from Accelerate Inc., Correvio, Pfizer and Shionogi.

### Funding

This study was funded by an unrestricted research grant by Accelerate Diagnostics Inc., USA. The sponsor had no influence on the design of the study and on the published results. 

### Authors’ contributions

KFB and MW were responsible for the study design and undertook the case reviews. WH was performing all statistical analyses and was responsible for the data collection. LS supported with literature research, proof-reading, conducting tables and drawings for this publication.

### Acknowledgements

The special thanks of the authors go to the teams of the ICU in the hospital as well as to the lab team that readily adopted this new diagnostic test and achieved a good overall adherence on testing the candidates for the Pheno™ system. Further we thank Prof. Reinier Mutters from Marburg who undertook an independent review of the findings from the case reviews.

### Authors’ information

KFB is a member of the Paul-Ehrlich Gesellschaft (PEG) and the responsible editor of the current guidelines for initial intravenous antimicrobial treatment of severe infections. He is a specialist for internal medicine, intensive care medicine, infectious diseases and also a certified antimicrobial stewardship (in Germany ABS) expert.

MW is a surgeon by training, has longstanding experience in intensive care and is an antimicrobial stewardship expert. He also has a history in hospital management and health economy. Currently he is a Professor of hospital management at the Medical School Hamburg (MSH). He is a member of PEG, where he chairs the working group ‘economic aspect of anti-infective therapy’.

WH is a MD (internal medicine) and holds a degree in medical informatics. He is an expert in database design, data management and statistical analysis.

LS holds a B.Sc. in biomedical engineering, is a student and currently working on his master thesis in the field of medical process management. 

## Figures and Tables

**Table 1:**
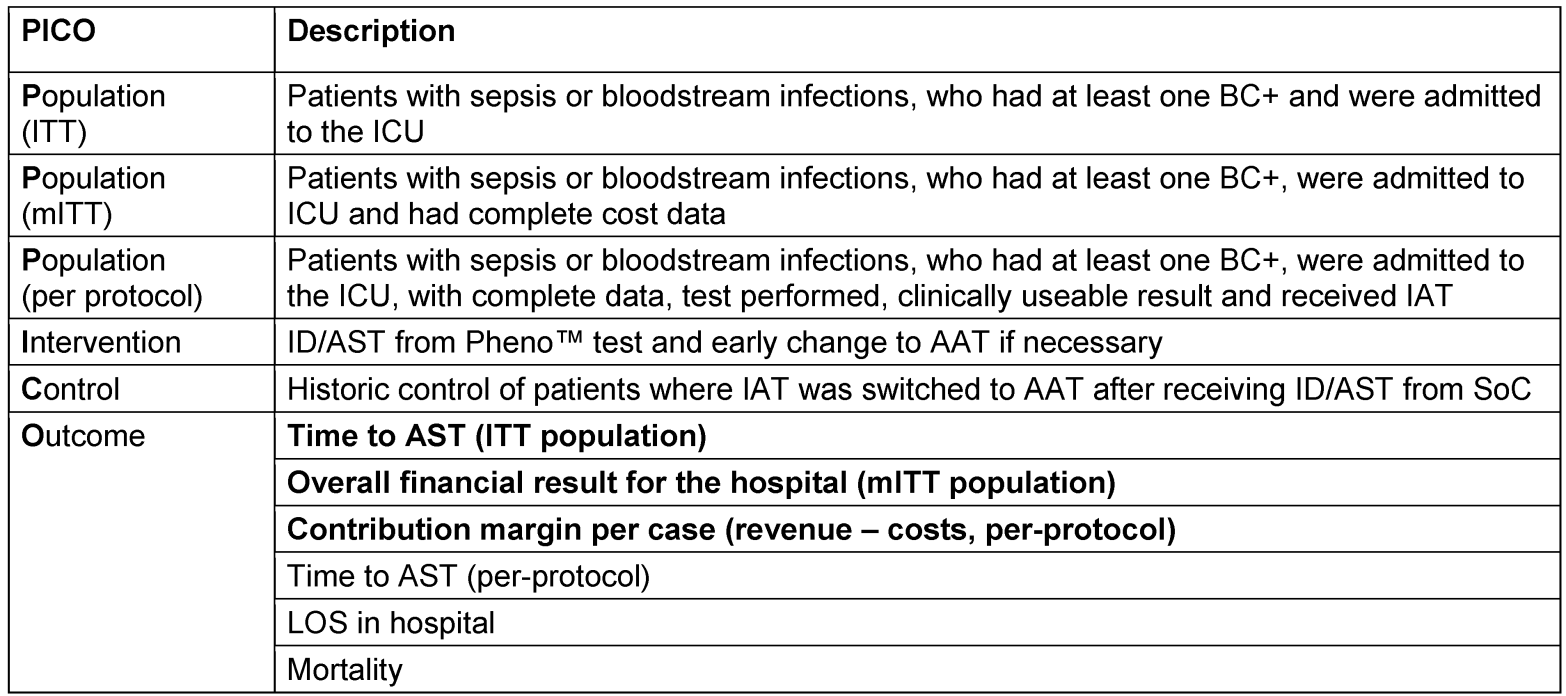
Study design according to the PICO principle

**Table 2:**
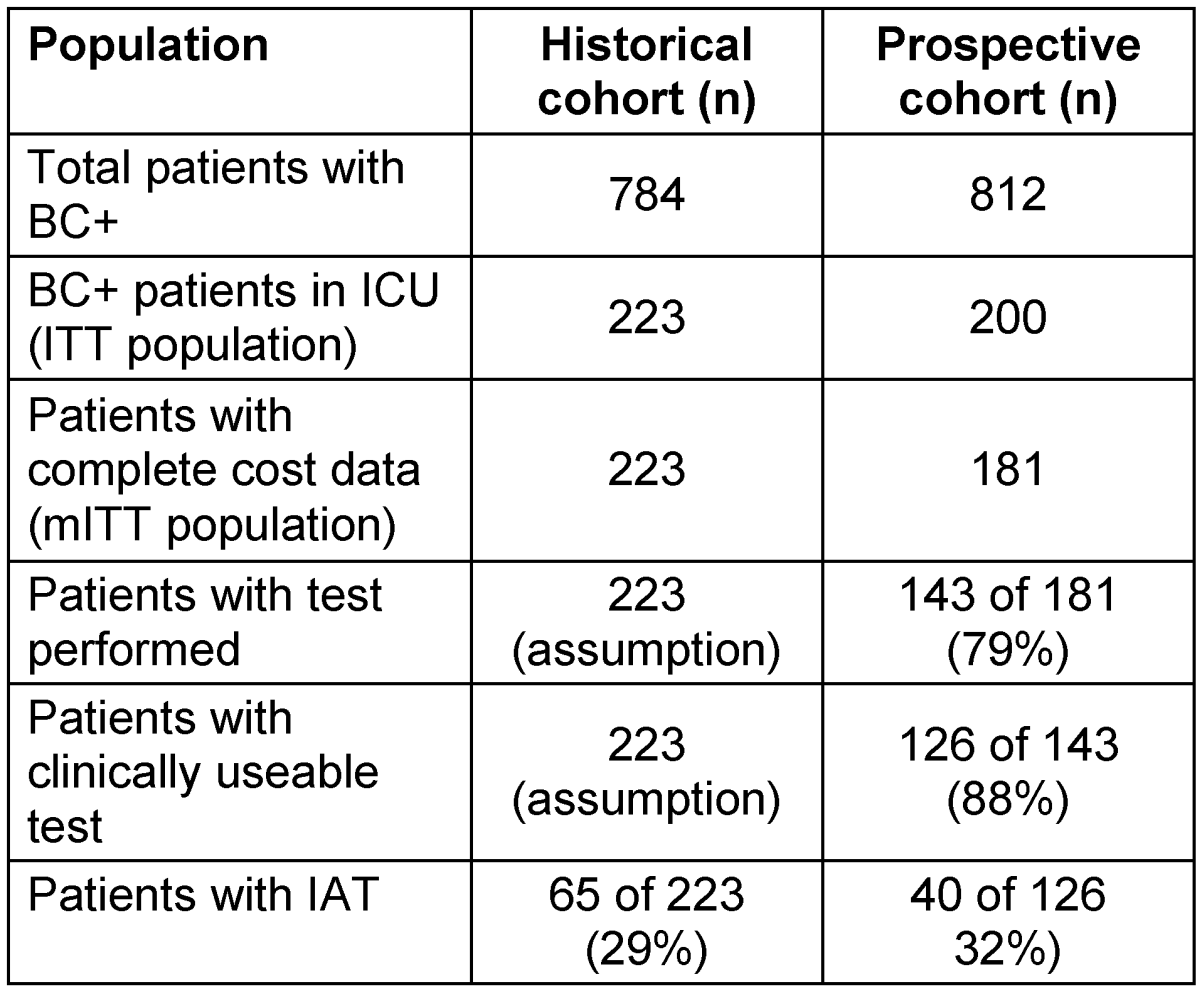
Patient selection and assignment to analysis populations

**Table 3:**
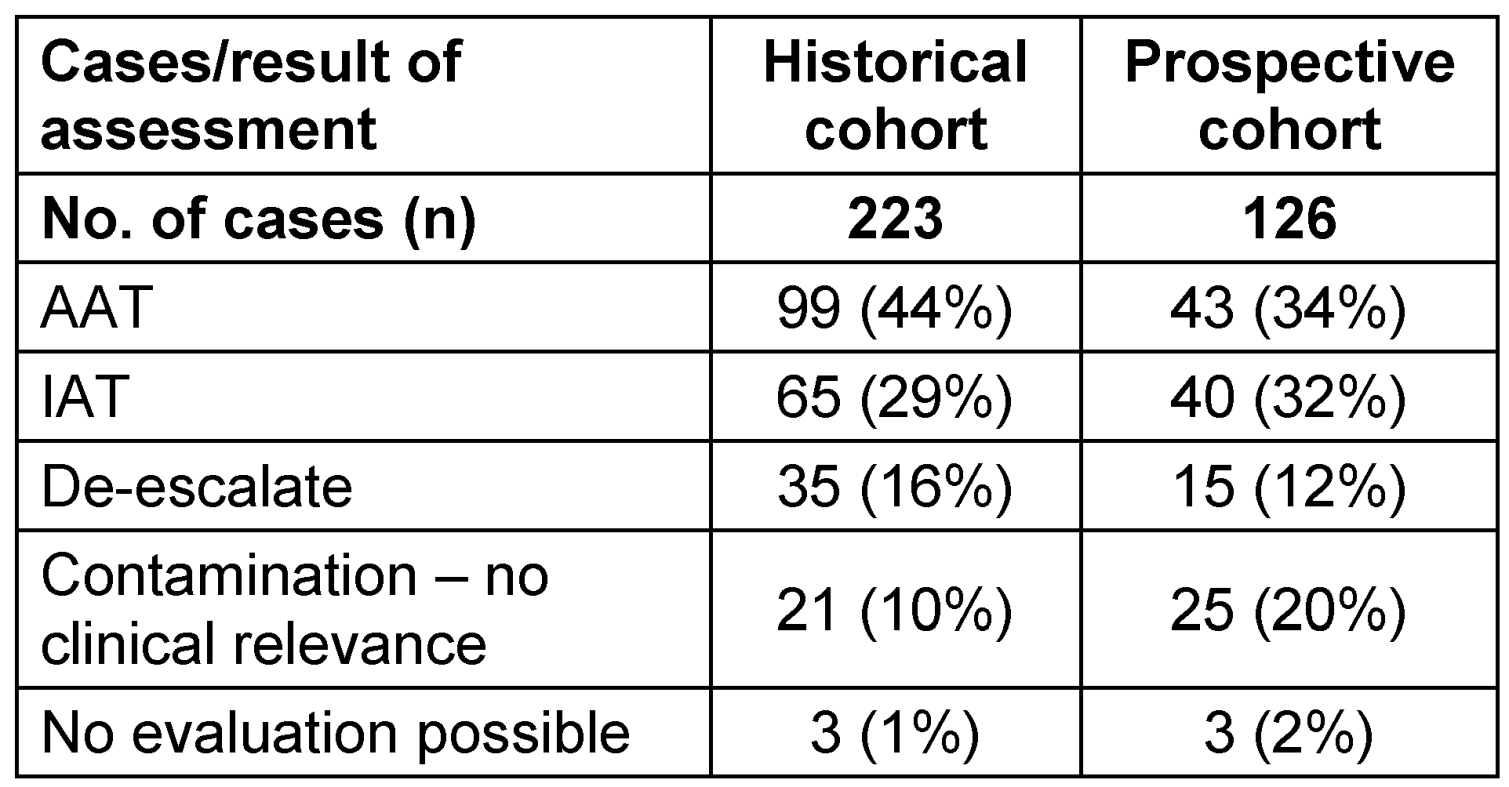
Results of the AT assessment in both cohorts

**Table 4:**
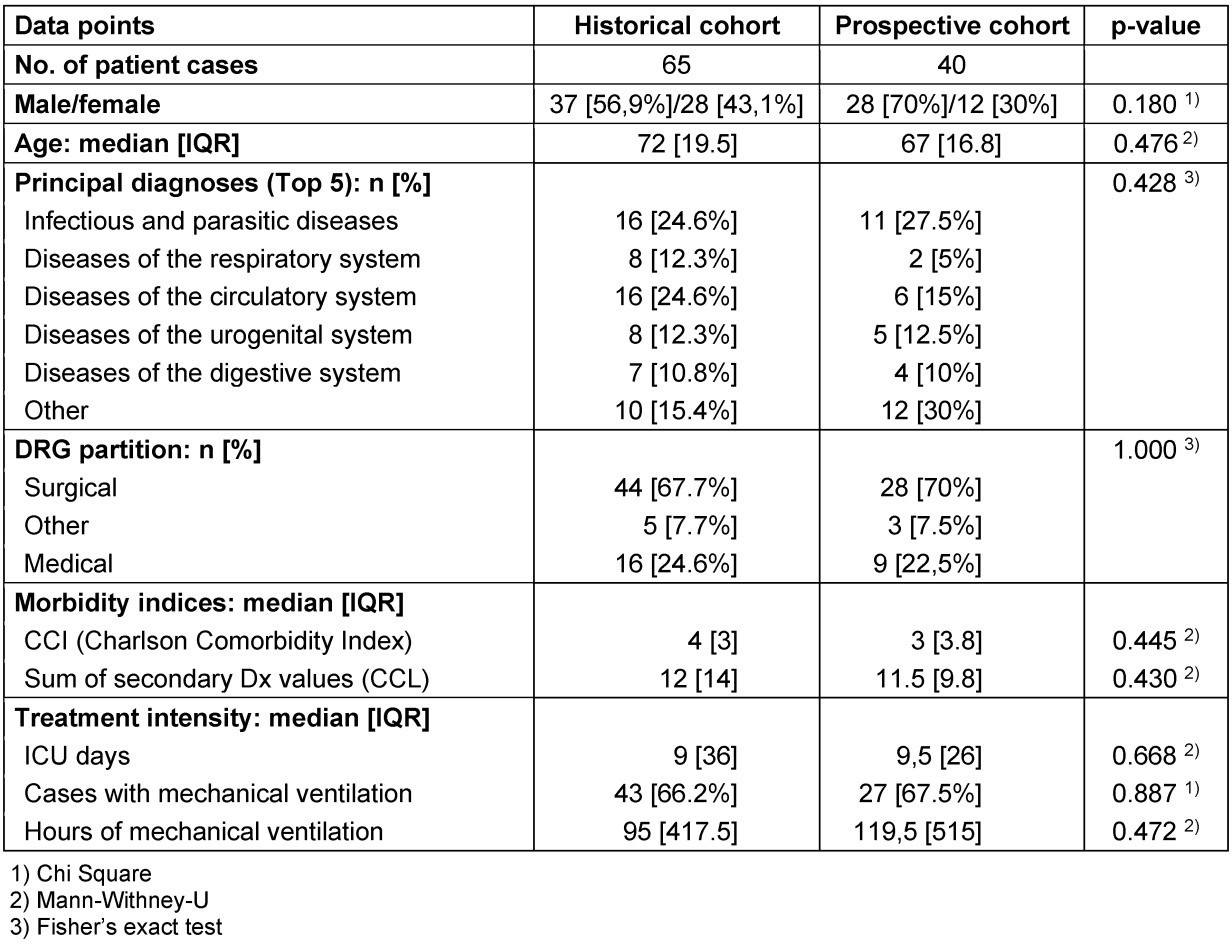
Baseline characteristics with no significant differences between the groups

**Table 5:**
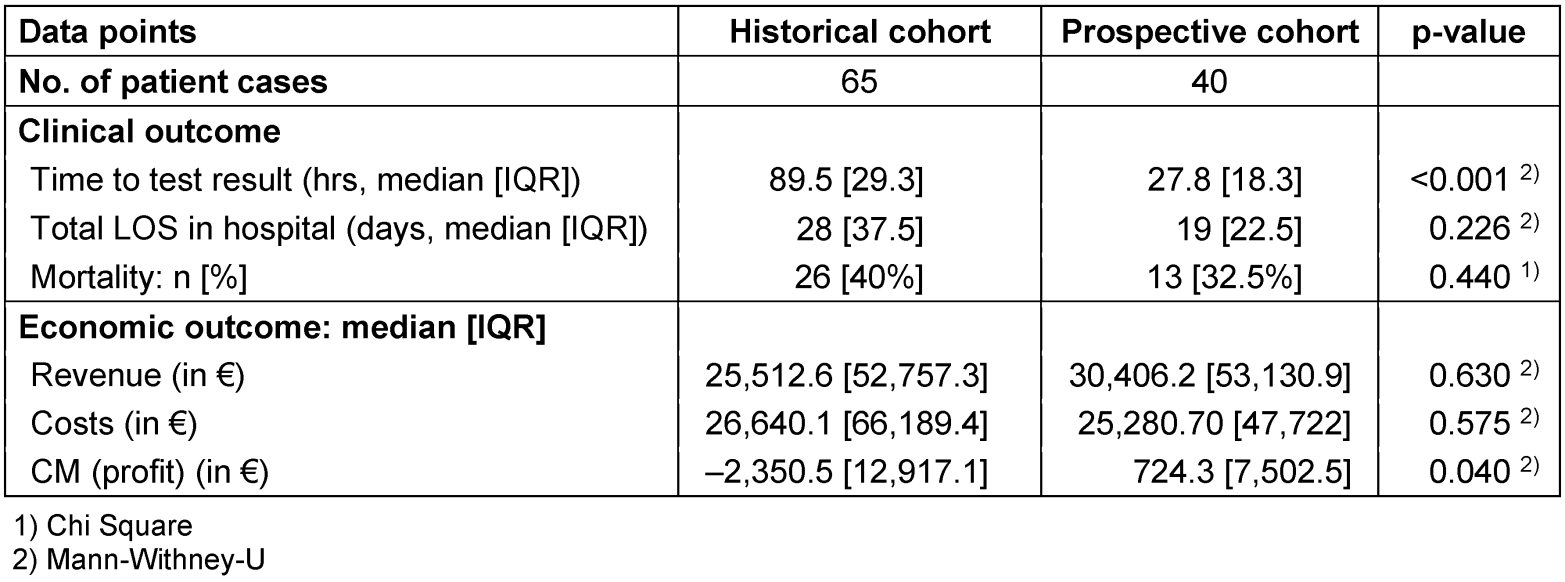
Clinical and economic outcomes of patients in the PP population

**Table 6:**
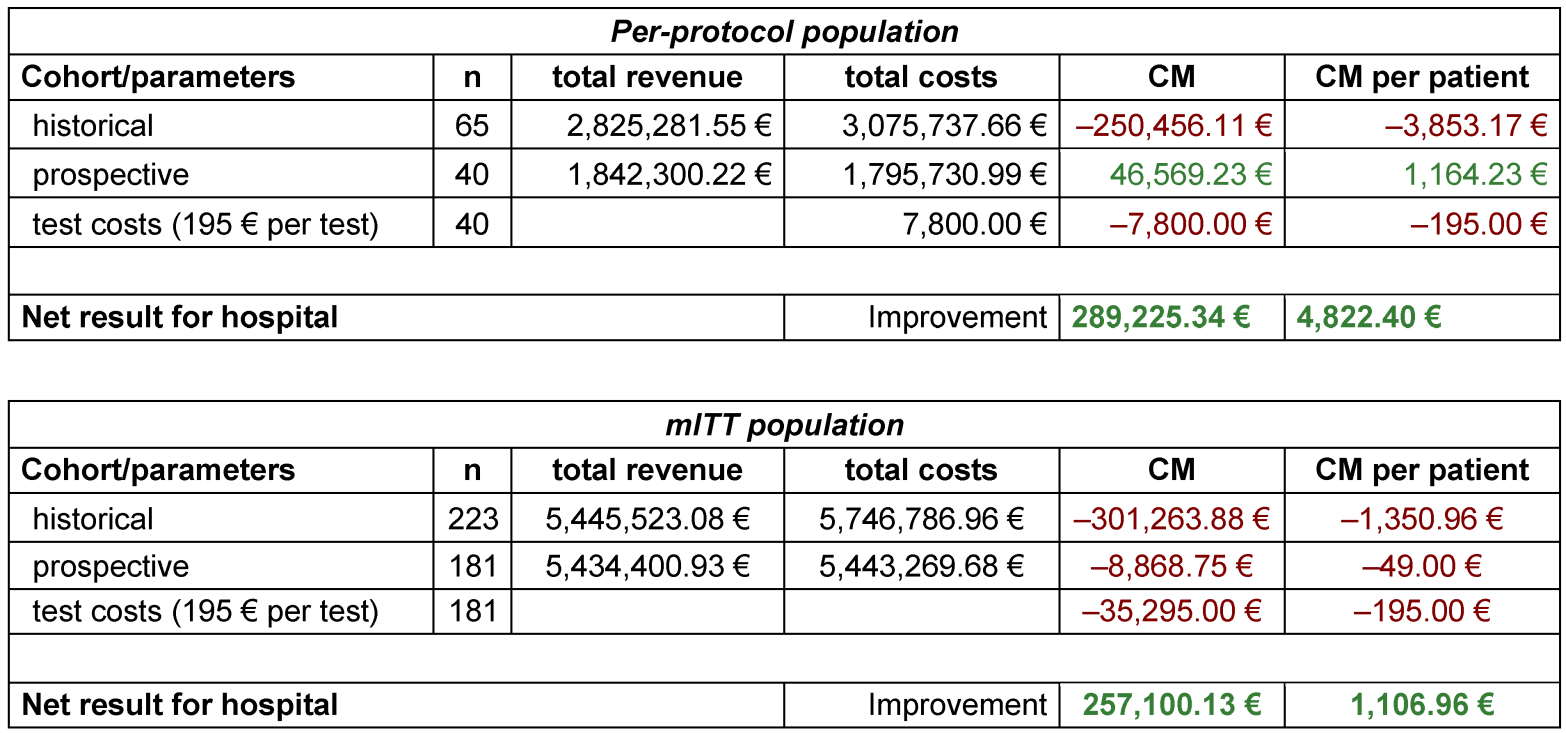
Total economical result
